# Factors Associated With the Intention to Begin Physical Activity Among Inactive Middle-Aged and Older Adults

**DOI:** 10.1177/10901981211030867

**Published:** 2021-08-20

**Authors:** Ariane S. Massie, Heather Johnston, Daniel Sibley, Brad A. Meisner

**Affiliations:** 1 York University, Toronto, Ontario, Canada

**Keywords:** behavior and behavior mechanisms, health promotion, life course, mental processes, motivation

## Abstract

Factors that affect physical activity (PA) behavior change are well established. Behavioral intention is a strong psychological predictor of behavior; however, there is less research on the factors that affect the *intention* to increase PA participation specifically, especially among adults in mid and later life who are inactive. Using data from the Canadian Community Health Survey, which was informed by the transtheoretical model (TTM), this study investigated the relationships between a range of demographic and biopsychosocial factors with the intention to become physically active among 1,159 inactive adults aged 40 years and older. Comparisons were made between participants reporting the intention to begin PA in the next 30 days (TTM Preparation; *n* = 610), 6 months (TTM Contemplation; *n* = 216), or not at all (TTM Precontemplation; *n* = 333). First, multinomial logistic regression identified age, sex, ethnicity, education, restriction of activities, self-perceived health, and community belonging as factors significantly associated with 30-day PA intention, while age and ethnicity were significantly associated with 6-month PA intention, compared with those reporting no intention. Second, binary logistic regression revealed that education was the only factor that differentially associated with intention timeframe as participants with lower levels of education were less likely to report PA intention in 30 days compared with 6 months. Findings demonstrate key demographic, biopsychosocial, and temporal factors that warrant consideration for tailored PA promotion programs that aim to effectively address the constraints and barriers that negatively influence PA intention among middle-aged and older adults.

Research demonstrates that one’s intention to be physically active is significantly and positively associated with subsequent PA participation (Chatzisarantis et al., 2019). A meta-analysis examining the relationship between PA intention and behavior found that 54% of people who had the intention to engage in PA went on to become physically active (Rhodes & de Bruijn, 2013). While this is a seemingly low percentage, that same meta-analysis found that only 2% of those who did not have the intention to engage in PA subsequently changed their behavior. Therefore, people who have the intention to become active have significantly greater odds of behavioral change than those who do not. This evidence positions PA intention as an important and useful precursor to PA participation (Sheeran, 2002; Webb & Sheeran, 2006).

Intention is such a strong psychological predictor of behavior that it is incorporated into behavioral change theories. For instance, the stages-of-change transtheoretical model (TTM) stipulates that when adopting or changing a behavior, such as PA, individuals move through a series of stages, which include (a) precontemplation (i.e., no intention to change); (b) contemplation (i.e., intention to change in the long term); (c) preparation (i.e., intention to change in the short term); (d) action (i.e., engagement in the new behavior); and (e) maintenance (i.e., sustained participation in the behavior over time; Prochaska & DiClemente, 1983). Within the TTM tenets, behavioral intention is the driving motivational force toward action. People in the contemplation and preparation stages are similar as both share the intention to change a behavior in the near future, although within different timeframes (e.g., 6 months and 30 days, respectively).

It is important to acknowledge that the pathway from PA intention to action is complex and can be constrained by a range of internal and external personal factors—particularly in mid and later life (Alley et al., 2018). This effect (or lack thereof) is known as the intention–action gap (Rhodes & de Bruijn, 2013; Sheeran & Webb, 2016). However, from a lifespan perspective, there is an observed decrease in the intention–action gap with age (Hagger et al., 2002). Therefore, although middle-aged and older adults are significantly less likely than younger adults to have the intention to engage and to participate in recommended levels of PA (Alley et al., 2018; Crombie et al., 2004; Harvey et al., 2015), those in mid and later life who do have PA intention are more likely to transition to PA participation (Hagger et al., 2002). This trend indicates that PA intention has a greater influence on PA participation among middle-aged and older adults than others earlier in their lifespan.

To date, the vast majority of PA and aging research has investigated the multidisciplinary associates of either PA participation or inactivity rather than PA intention (Fisher et al., 2018; John et al., 2020). There is no existing research that discerns what factors are associated with PA intention among inactive middle-aged and older adults who are either contemplating or preparing for PA. Identifying the associates of these two TTM stages of change is a promising research endeavor as this evidence can be used to develop new or existing PA interventions. For example, by informing tailored recruitment and customized content implementation strategies for PA program participants who are specifically ready for change based on when this change is expected to occur (Garber et al., 2008; Prince et al., 2016). Indeed, there are pronounced effects of, and adherence to, PA programs that explicitly consider and support PA intention among middle-aged and older adults within intervention designs, compared with interventions that do not emphasize PA intention (Greaney et al., 2008).

Using data from a sample of inactive middle-aged and older adults, the objectives of this study were twofold. First, to examine the potential association of a series of internal and external multidisciplinary factors with the intention to begin PA in either the next 30 days or 6 months compared with the intention to remain inactive. Second, to determine which of these factors are differentially associated with the intention to begin PA in the next 30 days compared with the next 6 months.

## Method

### Study Design and Data Collection

This study analyzed data from the Canadian Community Health Survey (CCHS). The CCHS is a validated and reliable population-based, government-administered, cross-sectional survey. The CCHS questionnaire was developed by specialists from Statistics Canada (2020) with other federal, provincial, and territorial governmental departments. Data are collected from community-dwelling individuals aged 12 years and greater on an annual basis. Random-sampling strategies are used to identify participants from all 10 provinces and 3 territories of Canada. The CCHS includes both core and optional content. Core content is mandatory across Canada, whereas optional content is selected by each province or territory depending on the identified priorities of the region. Data used for the current study were from the CCHS 2014 Annual Component, which was the only component to include optional data on PA intention selected by the province of British Columbia and the Northwest Territories. All demographic and biopsychosocial factors of the current study were core content. For more information about the CCHS, please refer to the online resources provided by Statistics Canada (2020).

### Sample

To achieve the research objective, participants were selected based on chronological age and the CCHS Physical Activity Index. This index classifies participants as active, moderately active, or inactive based on average daily energy expenditure equivalents of self-reported PA over the past 3 months. Specifically, participants were included in the analyses if they were (a) aged 40 years and greater at the time of data collection, and (b) classified as “inactive” according to the CCHS PA Index. The inactive classification represents individuals who have an average daily energy expenditure of less than 1.5 kcal per kg of body weight, which is the equivalent to walking no more than 15 minutes per day. The final sample that met both criteria included 1,159 participants.

### Measures

#### Intention to Begin PA Outcomes

Participants were asked “Do you intend to increase your physical activity level in the next 30 days?” and “Do you intend to increase your physical activity level in the next six months?” Both items were answered with “yes” or “no” responses. These responses were combined to derive the two outcome variables for the current study. First, a three-group variable was created to classify participants who reported either (a) the intention to increase PA in the next 30 days (TTM Preparation; “yes” response for 30-day PA intention), (b) the intention to increase PA in the next 6 months (TTM Contemplation; “yes” response for 6-month PA intention), or (c) no intention to increase PA in either timeframe (TTM Precontemplation; “no” response for 30-day or 6-month PA intention; *n* = 1,159).

Second, a two-group variable was created to classify participants who reported either (a) the intention to increase PA in the next 30 days (TTM Preparation; “yes” response for 30-day PA intention) and (b) the intention to increase PA in the next 6 months (TTM Contemplation; “yes” response for 6-month PA intention) only (*n* = 826). This two-group variable separated those with PA intention from those without, which allowed for a temporal comparison of characteristics between participants with PA intention at two different time points in the analyses (i.e., TTM Preparation, 30-day PA intention *versus* TTM Contemplation, 6-month PA intention). Participants already engaged in PA (TTM Action and Maintenance) were not included in the analyses due to the selection of “inactive” cases only.

#### Demographic and Biopsychosocial Predictors

Factors were selected from the measures available in the CCHS based on previous PA participation research, as cited below. Age, sex, ethnicity, education, and income were included as demographic predictor variables to explore if and how these factors were significantly associated with PA intention (e.g., Meisner et al., 2010; Rhodes & Quinlan, 2015; Trost et al., 2002). Age was categorized in 10-year groups (40–49 years, 50–59 years, 60–69 years, or 70 years and older). Sex and ethnicity were self-reported based on CCHS dichotomies of female or male and person of color or White. Education was measured as the highest personal level of formal education attained (less than postsecondary, some postsecondary, or postsecondary). Income included participants’ total annual household income measured in $20,000 increments (less than $20,000; $20,000-$39,999; $40,000-$79,999; or $80,000 and greater).

In terms of the biopsychosocial predictor variables, the diagnosis of a chronic health condition (yes or no) and the restriction of activities due to that chronic condition (often, sometimes, or never) were included as biological factors (e.g., Boyle et al., 1998; Kamil-Rosenberg et al., 2019). Self-perceived health (high, moderate, or low) and self-perceived life stress (high, moderate, or low) were included as psychological factors (e.g., Steptoe et al., 2015; Stults-Kolehmainen & Sinha, 2014). Self-perceived health was originally measured in the CCHS on a 5-point scale (i.e., poor, fair, good, very good, and excellent), which was transformed into low (i.e., poor and fair), moderate (i.e., good), and high (i.e., very good and excellent) levels. Self-perceived life stress was also originally measured on a 5-point scale (i.e., not at all stressful, not very stressful, a bit stressful, quite a bit stressful, and extremely stressful), which was transformed into low (i.e., not at all and not very stressful), moderate (i.e., a bit stressful), and high (i.e., quite a bit and extremely stressful) levels. Relationship status and sense of community belonging were included as social factors (e.g., Morgan et al., 2019; Ross & Searle, 2019). Relationship status was classified in terms of participants who had a partner (common-law and married) and those who did not (divorced, separated, widowed, and single/never married). Sense of community belonging included participants who did and did not report a strong sense of community belonging.

### Statistical Analyses

Descriptive statistics were used to characterize the sample. Multinomial logistic regression was used to estimate the associations between the demographic and biopsychosocial factors and the three-group PA intention outcome. Two sets of odds ratio (*OR*) and 95% confidence interval (CI) statistics were obtained to represent associations for participants reporting the intention to begin PA in (a) the next 30 days (TTM Preparation) compared with those reporting no PA intention (TTM Precontemplation), and (b) the next 6 months (TTM Contemplation) compared with reporting no PA intention (TTM Precontemplation). Binary logistic regression was used to compare demographic and biopsychosocial associations between the 30-day and 6-month intention timeframes directly. This set of *OR*s and 95% CIs represent factors associated with the intention to begin PA in the next 30 days compared with the next 6 months. Multivariate adjusted results of the full models are reported below. Analyses were performed using SPSS (IBM Corp. Released 2019. IBM SPSS Statistics for Windows, Version 26.0. Armonk, NY).

## Results

Descriptive statistics are presented in Table 1. Most participants reported the intention to begin PA in either timeframe (71.3%, *n =* 826), but more so in the next 30 days (52.6%, *n* = 610) than next 6 months (18.6%, n = 216). The sample mode for age was 70 years and greater (29.6%, *n* = 343). More than half were female (61.3%, *n* = 711) and had completed postsecondary education (53.7%, *n* = 616). Most participants self-identified as White (82.0%, *n* = 920). For annual household income, the sample mode was between $40,000 and $79,999 (31.3%, *n* = 363). The majority had at least one chronic condition (81.6%, *n* = 946) and some level of restriction to their activities (64.5%, *n* = 748). The sample was distributed across high, moderate, and low ratings of self-perceived health (Range: 30.1%, *n* = 358 to 37.7%, *n* = 437) and self-perceived life stress (Range: 23.3%, *n* = 269 to 39.4%, *n* = 454). Over half had a relationship partner (54.0%, *n* = 623), and a strong sense of community belonging (63.5%, *n* = 724).

**Table 1.** Descriptive Statistics for Overall and Stratified Sample by PA Intention.

Measure	Response category	Overall		No intention		30-day PA intention		6-month PA intention	
		*n*	%_valid_	*n*	%_valid_	*n*	%_valid_	*n*	%_valid_
Age, years	40–49	182	15.7	30	9.0	117	19.2	35	16.2
	50–59	293	25.3	60	18.0	176	28.9	57	26.4
	60–69	341	29.4	88	26.4	190	31.1	63	29.2
	70 and older	343	29.6	155	46.6	127	20.8	61	28.2
Sex	Male	448	38.7	140	42.0	217	35.6	91	42.1
	Female	711	61.3	193	58.0	393	64.4	125	57.9
Ethnicity	White	920	82.0	256	80.0	484	81.5	180	86.5
	Person of color	202	18.0	64	20.0	110	18.5	28	13.5
Highest level of education	Less than postsecondary	190	16.6	82	25.0	71	11.7	37	17.3
	Some postsecondary	340	29.7	93	28.4	169	28.0	78	36.4
	Postsecondary	616	53.7	153	46.6	364	60.3	99	46.3
Annual household income, $	Less than 20,000	164	14.2	63	18.9	77	12.6	24	11.1
	20,000–39,999	318	27.5	121	36.4	139	22.8	58	26.9
	40,000–79,999	363	31.3	91	27.3	197	32.4	75	34.7
	80,000 and greater	313	27.0	58	17.4	196	32.2	59	27.3
Chronic condition	No	213	18.4	50	15.0	129	21.1	34	15.7
	Yes	946	81.6	283	85.0	481	78.9	182	84.3
Restriction of activities	Sometimes	299	26.0	65	19.6	175	28.9	59	27.4
	Often	449	39.0	176	53.2	186	30.7	87	40.5
	Never	404	35.0	90	27.2	245	40.4	69	32.1
Self-perceived health	High	348	30.1	75	22.6	213	34.9	60	27.8
	Moderate	437	37.7	112	33.7	240	39.4	85	39.3
	Low	373	32.2	145	43.7	157	25.7	71	32.9
Self-perceived stress	Low	430	37.3	137	41.8	215	35.3	78	36.1
	Moderate	454	39.4	120	36.6	249	40.9	85	39.4
	High	269	23.3	71	21.6	145	23.8	53	24.5
Relationship status	Without partner	531	46.0	173	52.4	258	42.4	100	46.3
	With partner	623	54.0	157	47.6	350	57.6	116	53.7
Community belonging	No	416	36.5	134	41.5	196	32.5	86	40.2
	Yes	724	63.5	189	58.5	407	67.5	128	59.8
Total sample	*n*	1,159		333		610		216	

*Note*. Missing data for each measure were the following, *n* (%): Ethnicity = 37 (3.2%), highest level of education = 13 (1.1%), annual household income = 1 (0.1%), restriction of activities = 7 (0.5%), self-perceived health = 1 (0.1%), self-perceived stress = 6 (0.5%), relationship status = 5 (0.4%), and community belonging = 19 (1.6%). There were no missing data for age, sex, or chronic condition measures. PA = physical activity.

### Intention to Begin PA in Next 30 Days Versus No Intention

Presented in Table 2, three factors were positively associated with the intention to begin PA in the next 30 days compared with not having PA intention. Participants in the 40- to 49-year, 50- to 59-year, and 60- to 69-year age groups each demonstrated increased odds compared with those 70 years and older (*OR*_Range_ = 2.11–3.29, 95% CI_Range_ [1.44, 5.69]). Those self-identifying as White had increased odds than participants self-identifying as a person of color (*OR* = 1.68, 95% CI [1.13, 2.52]). Participants reporting both high and moderate levels of self-perceived health demonstrated increased odds compared with those with low self-perceived health (*OR*_Range_ = 1.46–1.57, 95% CI_Range_ [1.00, 2.45]). Additionally, four other factors were negatively associated with the intention to begin PA in the next 30 days. Males had decreased odds than females (*OR* = 0.66, 95% CI [0.48, 0.90]). Participants with less than postsecondary education had decreased odds compared with those with postsecondary levels (*OR* = 0.51, 95% CI [0.33, 0.77]). Participants whose activities were restricted often had decreased odds than those with no restriction (*OR* = 0.48, 95% CI [0.31, 0.73]). Those without a strong sense of community belonging had decreased odds compared with those with belonging (*OR* = 0.70, 95% CI [0.51, 0.95]).

**Table 2.** Multivariate Associations Between Factors and Either 30-Day or 6-Month PA Intention Compared With No PA Intention (*n* = 1,159).

Measure	Response category	30-day PA intention		6-month PA intention	
		*OR*_adjusted_	95% CI	*OR*_adjusted_	95% CI
Age, years	40–49	3.29	[1.91, 5.69]	2.78	[1.44, 5.38]
	50–59	3.07	[1.97, 4.77]	2.34	[1.37, 4.00]
	60–69	2.11	[1.44, 3.10]	1.62	[1.01, 2.61]
	70 and older	Referent		Referent	
Sex	Male	0.66	[0.48, 0.90]	0.91	[0.62, 1.33]
	Female	Referent		Referent	
Ethnicity	White	1.68	[1.13, 2.52]	2.06	[1.22, 3.50]
	Person of color	Referent		Referent	
Highest level of education	Less than postsecondary	0.51	[0.33, 0.77]	1.06	[0.64, 1.77]
	Some postsecondary	0.74	[0.52, 1.05]	1.28	[0.84, 1.96]
	Postsecondary	Referent		Referent	
Annual household income, $	Less than 20,000	0.85	[0.48, 1.50]	0.56	[0.27, 1.16]
	20,000–39,999	0.66	[0.42, 1.05]	0.70	[0.40, 1.24]
	40,000–79,999	0.94	[0.61, 1.43]	1.02	[0.61, 1.70]
	80,000 and greater	Referent		Referent	
Chronic condition	No	0.79	[0.50, 1.23]	0.74	[0.42, 1.29]
	Yes	Referent		Referent	
Restriction of activities	Sometimes	1.08	[0.70, 1.66]	1.09	[0.64, 1.84]
	Often	0.48	[0.31. 0.73]	0.68	[0.41, 1.14]
	Never	Referent		Referent	
Self-perceived health	High	1.57	[1.00, 2.45]	1.38	[0.80, 2.38]
	Moderate	1.46	[1.01, 2.13]	1.36	[0.86, 2.14]
	Low	Referent		Referent	
Self-perceived stress	Low	0.91	[0.59, 1.39]	0.86	[0.52, 1.45]
	Moderate	0.89	[0.60, 1.33]	0.91	[0.56, 1.48]
	High	Referent		Referent	
Relationship status	Without partner	1.02	[0.72, 1.44]	1.10	[0.73, 1.66]
	With partner	Referent		Referent	
Community belonging	No	0.70	[0.51, 0.95]	0.93	[0.64, 1.36]
	Yes	Referent		Referent	

*Note*. The outcome referent group was reporting no PA intention at either 30 days or 6 months. In terms of the TTM, 30-day PA intention represents the “Preparation” stage, 6-month PA intention represents the “Contemplation” stage, and no PA intention at either 30 days or 6 months represents the “Precontemplation” stage. PA = physical activity; TTM = transtheoretical model.

#### Intention to Begin PA in Next 6 Months Versus No Intention

Two factors were positively associated with the intention to begin PA in the next 6 months compared with not having PA intention, also found in Table 2. Similar to 30-day PA intention, participants in each of the three younger age groups demonstrated increased odds compared with the 70 years and older group (*OR*_Range_ = 1.62–2.78, 95% CI_Range_ [1.01, 5.38]). Furthermore, White participants had increased odds than people of color (*OR* = 2.06, 95% CI [1.22, 3.50]).

### Intention to Begin PA in Next 30 Days Versus Next 6 Months

Presented in Table 3, one factor was associated with the intention to begin PA in the next 30 days compared with next 6 months. Participants with less than or some postsecondary education both demonstrated decreased odds of 30-day PA intention compared with those with postsecondary levels (*OR*_Range_ = 0.48–0.57, 95% CI_Range_ [0.29, 0.82]).

**Table 3.** Multivariate Associations Between Factors and 30-Day PA Intention Compared With 6-Month PA Intention (*n* = 826).

Measure	Response category	*OR*_adjusted_	95% CI
Age, years	40–49	1.07	[0.61, 1.86]
	50–59	1.21	[0.75, 1.94]
	60–69	1.24	[0.79, 1.94]
	70 years and older	Referent	
Sex	Male	0.74	[0.53, 1.03]
	Female	Referent	
Ethnicity	White	0.80	[0.49, 1.28]
	Person of color	Referent	
Highest level of education	Less than postsecondary	0.48	[0.29, 0.79]
	Some postsecondary	0.57	[0.39, 0.82]
	Postsecondary	Referent	
Annual household income, $	Less than 20,000	1.51	[0.78, 2.93]
	20,000–39,999	0.98	[0.59, 1.65]
	40,000–79,999	0.93	[0.61, 1.44]
	80,000 and greater	Referent	
Chronic condition	No	1.09	[0.67, 1.76]
	Yes	Referent	
Restriction of activities	Sometimes	0.98	[0.63, 1.53]
	Often	0.67	[0.42, 1.06]
	Never	Referent	
Self-perceived health	High	1.11	[0.68, 1.81]
	Moderate	1.07	[0.70, 1.63]
	Low	Referent	
Self-perceived stress	Low	1.03	[0.64, 1.65]
	Moderate	1.00	[0.65, 1.54]
	High	Referent	
Relationship status	Without partner	0.94	[0.65, 1.37]
	With partner	Referent	
Community belonging	No	0.72	[0.51, 1.02]
	Yes	Referent	

*Note*. The outcome referent group was reporting 6-month PA intention. In terms of the TTM, 30-day PA intention represents the “Preparation” stage and 6-month PA intention represents the “Contemplation” stage. PA = physical activity; TTM = transtheoretical model.

## Discussion

In a large sample of inactive middle-aged and older adults, this study explored a set of biopsychosocial factors to discern their potential association with the intention to begin PA in the next 30 days or 6 months. Using data informed by the TTM, it was identified that, compared with those with no intention to participate in PA (Precontemplation): (a) participants with the intention to begin PA in the next 30 days (Preparation) were more likely to be relatively younger (i.e., middle-aged), female, White, with at least some postsecondary education, without frequent restrictions to their activity, and with at least moderate levels of self-perceived health and a strong level of community belonging; and (b) participants with the intention to begin PA in the next 6 months (Contemplation) were more likely to be relatively younger and White. This study also identified that education was the only one of these factors to be differentially associated with the likelihood of being in the preparation stage than the contemplation stage. It is important to note that these multivariate results were adjusted to control for the confounding effects of all factors included in the full models. Therefore, these findings represent independent mutually exclusive associations.

Two factors, age and ethnicity, were associated with the intention to begin PA in both 30-day and 6-month timeframes. Regarding age, middle-aged participants had greatest odds of having PA intention and, as age increased, odds decreased. This finding coincides with lifespan PA trends that demonstrate a negative association between chronological age and PA participation (Hansen et al., 2019; Shaw & Spokane, 2008; van der Zee et al., 2019). Not only was age significantly associated with both PA intention timeframes, but these associations demonstrated the largest effect sizes across all factors. Therefore, chronological age could be deemed one of the strongest predictors of PA intention. As a result, programs that aim to promote PA participation among older adults would benefit from strategies that motivate PA intention prior to behavior. Such interventions could focus on immediate benefits of PA (e.g., sensory experiences, enjoyment, and socialization; Phoenix & Orr, 2014) as some older adults, particularly in late life, may not be motivated by the long-term benefits of PA (e.g., improved physical health outcomes; Boulton et al., 2018). Additionally, PA messages should foster positive and realistic representations of older adults engaging in PA to help bolster PA intention and participation (Massie & Meisner, 2019).

Pertaining to ethnicity, those identified as White were more likely to have PA intentions than participants identified as people of color. This finding is supported by previous research that shows lower levels of individual- and group-based PA participation among people of color compared to Whites (Shaw & Spokane, 2008). These ethno-racial differences can be attributed to distinctions of culturally preferred modes of PA and to the availability or accessibility of preferred modes of PA (Corral, et al., 2012; Hawes et al., 2019), which in turn may reduce PA intentions. Future PA interventions must recognize and support culturally preferred modes of PA as well as address PA barriers associated with racism (Koshoedo et al., 2015). These objectives can be achieved through culturally aware community consultation on preferred antiracist PA equity and inclusion strategies. For effective consultation, implementation, and evaluation of such tailored PA programs, greater attention must be drawn to the selection of PA measurement tools (Kriska, 2000). PA participation is often underreported among people of color when such outcome measures are not culturally sensitive (Kriska & Rexroad, 1998); however, when definitions of PA are broadened, the proportion of people of color classified as “physically active” significantly increases (Brownson et al., 2000). As such, it may be possible that the PA measures used create CCHS PA Index may not be culturally sensitive enough to accurately classify people of color as active, moderately active, or inactive or to completely capture ethno-racial differences in PA outcomes.

In addition to age and ethnicity, other demographic factors were associated with 30-day PA intention. Males were less likely than females, which is a finding consistent with Varma et al. (2017) who found that men tend to reduce their PA levels earlier in life and engage in lower overall levels of PA in later life than women. However, other studies show that men typically experience fewer barriers and report higher levels of PA (Hurley et al., 2014; Shaw & Spokane, 2008). It may be possible that male participants did not report having the intention to increase their PA if they perceived their current PA participation levels to be unconstrained and sufficient. The discrepancy in previous literature and this current study highlight the necessity of further understanding the relationship between age, PA intention, sex, and/or gender when developing PA programs, aligning with global priorities to increase inclusion for all and reduce inequalities for PA participation (World Health Organization, 2018).

Education was another significant demographic factor associated with greater likelihoods of having the intention to begin PA in the next 30 days compared with both no PA intention and 6-month PA intention. Represented in the multinomial and binary logistic regression results, participants with less than or some postsecondary education were less likely to have PA intention than participants who completed secondary education. These findings match previous research that shows education is a significant predictor of PA participation in later life (Jenkin et al., 2017; Shaw & Spokane, 2008), and that educational attainment is a moderating factor of the relationship between PA intention and PA participation as the PA intention–action gap is smaller among people with higher levels of education (Schüz et al., 2017). One explanation for these findings may be the influence of education on employment opportunities and occupational PA before versus after retirement. People with lower levels of education are more likely to be employed in physically demanding jobs and their PA levels, and perhaps PA intentions, tend to diminish at retirement (Shaw & Spokane, 2008). Opposite observations are found among those with higher levels of education. This group tends to engage in greater PA levels after retirement (Shaw & Spokane, 2008), which would be reflected in their retirement PA intentions. Strategies that aim to promote PA intention and PA participation could be developed in ways that consider educational and life course differences and preferences.

In terms of the biopsychosocial factors, although reporting a chronic condition itself was not related to PA intention, having frequent restriction of activities due to a chronic condition was a significant factor associated with 30-day PA intention. The distinction between these two factors is important as it situates functional limitation as a larger constraint to PA intention rather than the mere presence of a chronic condition. Functional limitations can be comprised of multiple factors (e.g., biological changes in musculoskeletal function and structures, injury, pain, fear-avoidance, etc.). However, it is well established that these changes, while commonly associated with aging, can be attenuated through PA (Papa et al., 2017). Therefore, PA programs should not only accommodate a range of functional ability but also address risks and misconceptions about PA that may influence fear or avoidance of activity (Denkinger et al., 2015). Aligning with previous predictors and factors for PA participation (Paterson & Warburton, 2010), this finding highlights the need for universal and inclusive design of PA programs that accommodate a range of functional abilities in mid and later life which in turn may be more welcoming to older adults’ varying intentions to change.

There was also a significant association found for self-perceived health. Compared with participants with low self-perceived health, those with moderate and high levels were significantly more likely to have 30-day PA intention. Although there is no existing research that considers self-perceived health as an associate of PA intention among middle-aged and older adults, this psychological factor is shown to associate with PA participation in previous studies focusing on mid and later life (Meisner et al., 2017; Moore et al., 2012). This result implies that the group of people who may benefit from PA the most, those reporting low levels of self-perceived health, is not demonstrating an increased intention to begin PA. This result is independent of the presence of a chronic condition, restrictions on activities due to that condition, and all other factors included in the multivariate analyses.

Last, the strong sense of community belonging was significantly associated with 30-day PA intention. This finding reflects previous research that shows PA and physical recreation programming that meaningfully fosters social connections within the community and the sense of community belonging can facilitate adherence to, and enjoyment of, such programs among middle-aged and older adults (Hartley & Yeowell, 2015; Meisner et al., 2019; Yamada & Heo, 2016). Therefore, tailoring messages of PA programs and community physical recreation opportunities in ways that reinforce the sense of belonging and connection may be effective strategies to increase PA intention and subsequent PA participation (Dionigi & Lyons, 2017; Hutchinson et al., 2017).

### Limitations

The CCHS data used for this study comes from a reliable government source that allowed for a wide range of factors to be included in the analyses. However, the two PA intention outcome measures were optional content selected only by British Columbia and the Northwest Territories. Also, the CCHS is a cross-sectional survey, so the directionality of the observed associations cannot be determined. Furthermore, the secondary use of data results in no control over what variables are available and how items were measured or collected. For example, factors had to be selected from items available in the CCHS as indicators for demographic, biological, psychological, and social domains as done in previous secondary research (e.g., Baker et al., 2009; Meisner et al., 2017). Additionally, participants who reported having the intention to begin PA in the next 30 days were not asked whether they also had the intention to begin PA in the next 6 months.

## Conclusion

This study explored factors associated with PA intention among inactive middle-aged and older adults. Results demonstrate that a range of factors affect PA intention in mid and later life. Therefore, future research on PA intention and aging should adopt a holistic view, which considers demographic and biopsychosocial factors, to understand how different personal and social characteristics result in either an increased or decreased likelihood of readiness to begin PA. Another contribution of this study is that the CCHS data were guided by the TTM and findings highlight the importance of studying the temporal dimensions of PA intention as well. Factors associated with short-term PA intention (i.e., 30 days; TTM Preparation) were different than those associated with long-term PA intention (i.e., 6 months; TTM Contemplation) compared with each other and compared with having no intention (TTM Precontemplation). The TTM provides a framework with substantial empirical support for certain PA intervention strategies being more effective at different times, for different people, depending on their stage of behavior change (Prochaska & DiClemente, 1983; Stuart & Nanette, 2007). Future work could also explore what factors are associated with PA intention compared with PA participation (TTM Action and Maintenance).

There are important implications of the current study on PA interventions, programs, and policies pertaining aging and older adults. Findings provide evidence that a “one-size-fits-all” approach to PA promotion in mid and especially later life is not an effective strategy. Rather, such efforts should purposely consider demographic, biopsychosocial, and temporal factors that affect PA intention and the readiness to transition from PA intention to PA participation among middle-aged and older adults. If homogenizing PA strategies are taken, this study demonstrates that it would be those with the greatest odds of being ready to begin PA would benefit. Based on the results, these individuals are already among the most privileged and advantaged groups for accessible PA opportunities (i.e., younger vs. older adults; White vs. people of color; higher vs. lower educated; etc.). Diversifying PA strategies advocate for more tailored interventions in mid and later life, and research shows that this approach is more effective at narrowing the intention–action gap and improving PA participation outcomes compared with general interventions (Kroeze et al., 2006; Marcus et al., 1998). This study, along with future PA intention research, will provide important and useful knowledge on PA and aging as researchers and practitioners continue to examine ways to more successfully promote middle-aged and older adults’ intention to participate in PA.
